# Metal artifact reduction techniques for single energy CT and dual-energy CT with various metal materials

**DOI:** 10.1259/bjro.20180045

**Published:** 2019-07-08

**Authors:** Daisuke Kawahara, Shuichi Ozawa, Kazushi Yokomachi, Toru Higaki, Takehiro Shiinoki, Akito Saito, Tomoki Kimura, Ikuno Nishibuchi, Ippei Takahashi, Yuuki Takeuchi, Nobuki Imano, Katsumaro Kubo, Masayoshi Mori, Yoshimi Ohno, Yuji Murakami, Yasushi Nagata

**Affiliations:** 1 Radiation Therapy Section, Division of Clinical Support, Hiroshima University Hospital, Hiroshima, 734-8551, Japan; 2 Medical and Dental Sciences Course, Graduate School of Biomedical & Health Sciences, Hiroshima University, Hiroshima, 734-8551, Japan; 3 Department of Radiation Oncology, Institute of Biomedical & Health Sciences, Hiroshima University, Hiroshima, 734-8551, Japan; 4 Hiroshima High-Precision Radiotherapy Cancer Center, Hiroshima, 732-0057, Japan; 5 Departments of Diagnostic Radiology and Radiology, Hiroshima University, Hiroshima, 734-8551, Japan; 6 Department of Radiation Oncology, Graduate School of Medicine, Yamaguchi University, Yamaguchi, 755-0046, Japan; 7 Department of Radiation Oncology, Hiroshima Prefectural Hospital, Hiroshima, 734-8551, Japan

## Abstract

**Objective:**

The aim of the current study is to evaluate the effectiveness of reduction metal artifacts using kV-CT image with the single-energy based metal artefact reduction (SEMAR) technique by single-energy reconstruction, monochromatic CT and rED reconstructed by dual-energy reconstruction.

**Methods:**

Seven different metal materials (brass, aluminum, copper, stainless, steel, lead and titanium) were placed inside the water-based PMMA phantom. After DECT-based scan, the artefact index (AI) were evaluated with the kV-CT images with and without SEMAR by single-energy reconstruction, and raw-data based electron density (rED), monochromatic CT images by dual-energy reconstruction. Moreover, the AI with evaluated with rED and the converted ED images from the kV-CT and monochromatic CT images.

**Results:**

The minimum average value of the AI with all-metal inserts was approximately 80 keV. The AI without SEMAR was larger than that with SEMAR for the 80 kV and 135 kV CT images. In the comparison of the AI for the rED and ED images that were converted from 80 kV and 135 kV CT images with and without SEMAR, the monochromatic CT images of the PMMA phantom with inserted metal materials at 80 keV revealed that the kV-CT with SEMAR reduced the metal artefact substantially.

**Conclusion:**

The converted ED from the kV-CT and monochromatic CT images could be useful for a comparison of the AI using the same contrast scale. The kV-CT image with SEMAR by single-energy reconstruction was found to substantially reduce metal artefact.

**Advances in knowledge:**

The effectiveness of reduction of metal artifacts using single-energy based metal artefact reduction (SEMAR) technique and dual-energy CT (DECT) was evaluated the electron density conversion techniques.

## Introduction

CT imaging is an imaging modality that combines multiple X-ray projections taken from different angles to produce detailed 3D images of biological tissues. However, the process of acquiring images of patients using metallic hardware during CT scanning can result in severe artifacts that can obscure anatomic structures near the metal, and prevent the detection of features of interest such as pathologic lesions. Metallic artifacts are generated by photon starvation due to full absorption of the photon energy, which causes zero-transmission projections, or beam hardening caused by the absorption of low energy photon.^1^ Metal artifacts decreased the lesion identification for radiation diagnosis, and decreased target delineation and dose calculation accuracy for radiotherapy.^2,3^

Recently, new advanced metal artefact reduction techniques using single-energy and dual-energy methods have been introduced, and these techniques show promise in further reducing artifacts and improving the detection of pathologic lesions. Various metal artefact reduction (MAR) algorithms have been used in previous studies.^4,5^ Iterative MAR algorithms are mainly based on statistical models of image noise to improve image quality on each iteration of single-energy CT image reconstruction.^6^ The Toshiba Aquilion ONE single-energy CT scanner (Toshiba Corporation Medical Systems, Tokyo, Japan) uses a MAR algorithm called single-energy metal artefact reduction (SEMAR). SEMAR uses a modified sinogram inpainting technique to reduce metal artefact. This technique removes corrupt X-ray projections that traversed the metal and replace them with data from adjacent projections that did not traverse the metal.^7^ Moreover, the dual-energy CT (DECT) technique has been reported to effectively reduce metal artifacts.^5^ DECT can be used to obtain useful information such as the effective atomic number, electron density, and monochromatic CT numbers.^8^ A monochromatic CT image can be reconstructed from a pair of material density images and the corresponding mass attenuation coefficients.^9^ By eliminating the lower energy quanta, which is seen in the polychromatic spectrum of conventional CT scans, virtual monochromatic spectral imaging can reduce beam-hardening artifacts and metal artifacts.^10^ Furthermore, the Aquilion ONE is able to create a monochromatic CT image and the raw-data based electron density (rED) image.^11^ One advantage of this approach is the perfect alignment of the subsequent images, allowing material decomposition to be performed in the raw data that was sinogram based reconstructed. The metal artefact in these images was reduced using beam hardening correction on sinogram.^12^ The ED data has been used in treatment planning in radiation therapy. In general, the CT number in the scanned CT image is converted to electron density data using the CT-ED table created by the CT-ED phantom materials of well-known electron densities. The accuracy of the ED is a key component for dose calculations around the metal materials. Thus, the comparison based on the ED that is the same contrast scale between tissues is needed.

The aim of the current study is to evaluate the effectiveness of reduction metal artifacts using kV-CT image with the SEMAR technique by single-energy reconstruction, monochromatic CT and rED reconstructed by dual-energy reconstruction.

## methods and Materials

### Phantom and image acquition

**Figure 1.**
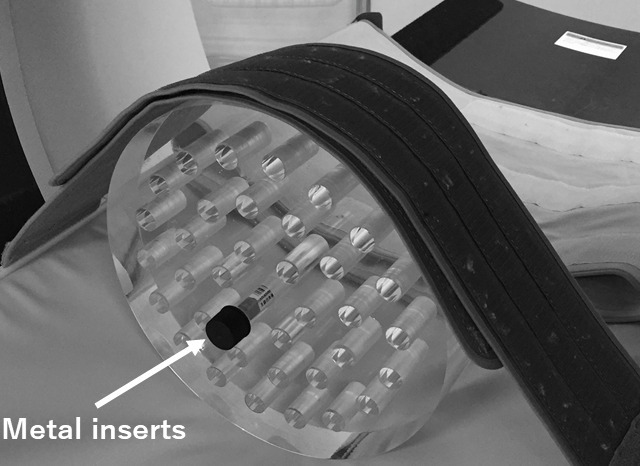
PMMA phantom that inserted metal materials in a syringe, which filled the water.

A water-based abdomen phantom was fabricated using polymethyl methacrylate (PMMA). Seven different metal materials (brass, aluminum, copper, stainless, steel, lead and titanium) were inserted in a syringe, and the gap between outer of the metal material and inner of the syringe was filled with the water. Then, it placed inside a water-based PMMA phantom that the diameter is 32 cm, as shown in Figure 1. DECT scans were performed at tube voltages of 80 and 135 kV. Exposures of 800 and 140 mA were used to minimize noise. The other scanning parameters were a rotation time of 1.0 s, slice thickness of 0.5 mm, and field of view of 400 mm. The middle of the acquired slices was analyzed in all cases. With the dual-energy reconstruction, rED and monochromatic CT images were reconstructed. Here, the monochromatic CT was reconstructed at energy of 40–130 keV. Moreover, the kV-CT images with and without SEMAR were reconstructed by single-energy reconstruction.

### Converted ED from kV-CT and monochromatic CT numbers with DECT

**Figure 2.**
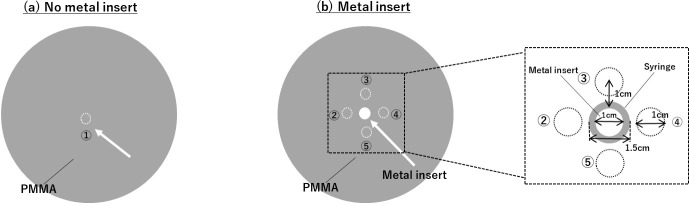
Electron Density Phantom that contained several tissue-equivalent inserts: lung (inhale), lung (exhale), adipose, breast, water, muscle, liver, trabecular bone (200 mg/cc hydroxyapatite), dense bone (800 mg/cc hydroxyapatite), and dense bone (1250 mg/cc hydroxyapatite).

A kV-CT to ED and monochromatic CT to ED calibration tables were created to convert the CT number to ED and the monochromatic CT number to ED. It was created based on an Electron Density Phantom Model 062M (Computerized Imaging Reference Systems: CIRS, Inc., Norfolk, VA), as shown in Figure 2. The phantom contained several tissue-equivalent inserts: lung (inhale), lung (exhale), adipose, breast, water, muscle, liver, trabecular bone (200 mg/cc hydroxyapatite), dense bone (800 mg/cc hydroxyapatite), and dense bone (1250 mg/cc hydroxyapatite), whose atomic compositions and densities are well-known and provided by the manufacturer. The kV-CT numbers and monochromatic CT numbers were converted to ED in the PMMA phantom inserted metal materials.

### Artifact propagation around the metal materials

**Figure 3.**
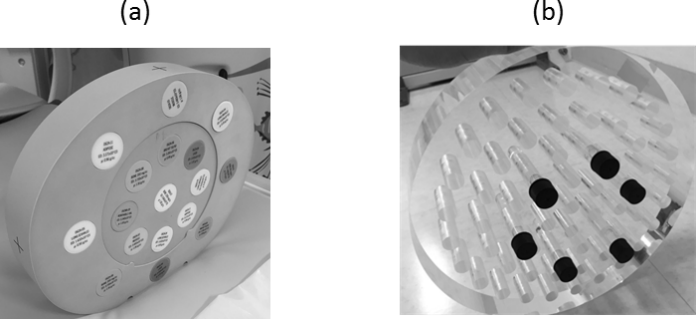
Method of measurement in the evaluation of the AI with the PMMA phantom that inserted metal materials that the diameter is 1 cm in a syringe that the diameter was 1.5 cm, which filled the water. The shape of the phantom and metal inserts were cylindrical column. The SDs were measured by creating a circular ROI with 1 cm diameter. The minimum distance of the center of ROI and the metal inserts was 1 cm.

Two assessments were conducted to evaluate the metal artifacts. One is to evaluate the AI for the kV-CT images with and without SEMAR, monochromatic CT images and rED images. The other to evaluate the ED-based AI for the rED image and the converted ED images from the kV-CT and monochromatic CT images. The images were analyzed with the software package ImageJ. The SD of the kV-CT numbers, monochromatic CT numbers and rED values were measured within a manually drawn region of interest (ROI) using ImageJ Circular ROIs which covered the maximum area within each of the materials. As shown in Figure 3, four ROIs for each image were drawn within 1 a cm area around the metal inserts. The artefact index (AI) was calculated using the measured SDs as follows^13^.

$$AI=\sqrt{{\left({SD}_{n}\right)}^{2}-{\left({SD}_{BG}\right)}^{2}}$$

where ${SD}_{n}$ and ${SD}_{BG}$ represent the SD values surrounding the metal inserts and background (BG) values, respectively. The *n* represents the region of the measurement (②-④). The ${SD}_{n}$ value was the average of the SD of ② to ④, ${SD}_{BG}$ value was the average of the SD of ①.

## Results

### AI with monochromatic images at 40–130 keV

**Figure 4.**
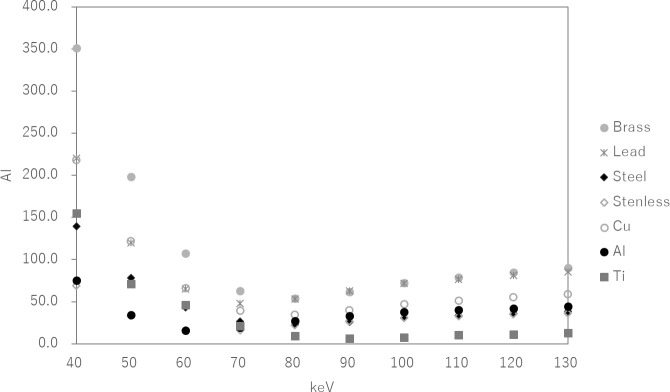
AI with monochromatic CTimages of the PMMA phantom that insertedmetal materials (brass, lead, steel, stainless, copper, aluminum and titanium)in the syringe, which filled the water, from 40 to 130 keV.

Figure 4 shows the AI with monochromatic CT images of PMMA phantom that inserted metal materials (brass, aluminum, copper, stainless, steel, lead and titanium) in a syringe that filled the water from 40 to 130 keV. The AI was highest at low-energy (40 keV) for all metal materials. At an energy of 70–90 keV, the artifacts around the inserted metal were reduced.

### Metal artefact for single-energy and dual-energy reconstruction

**Figure 5.**
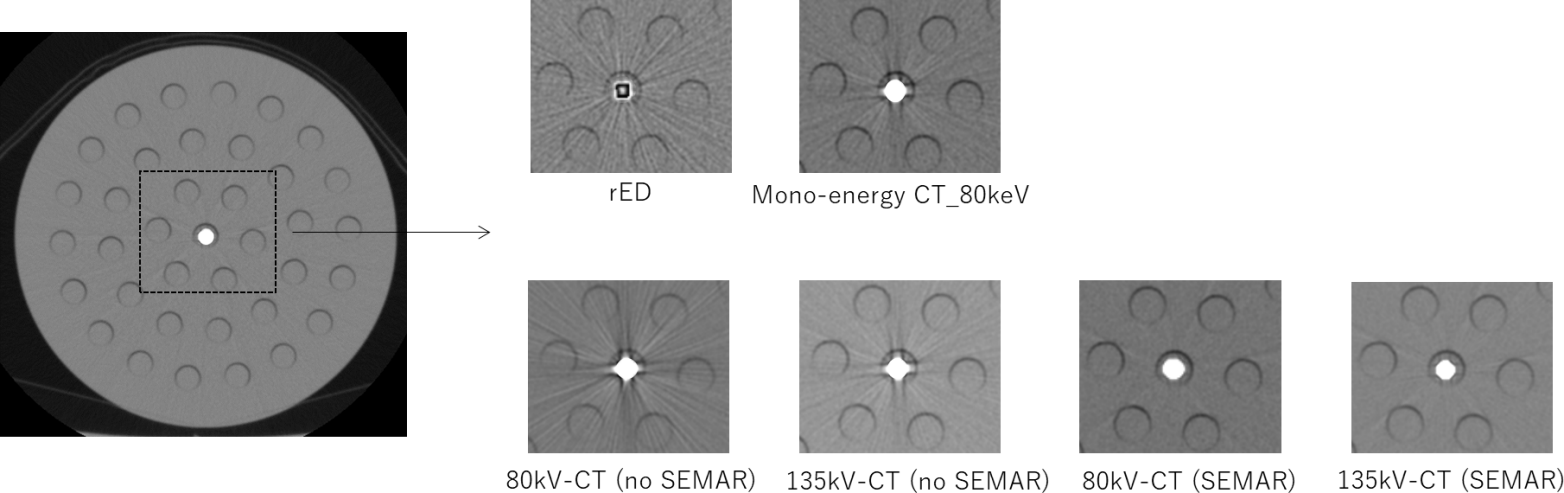
The rED, monochromatic energy CT at 80 keV and single energy CT with and without SEMAR at 80 kV and 135 kV using brass in the syringe, which filled the water.

**Figure 6.**
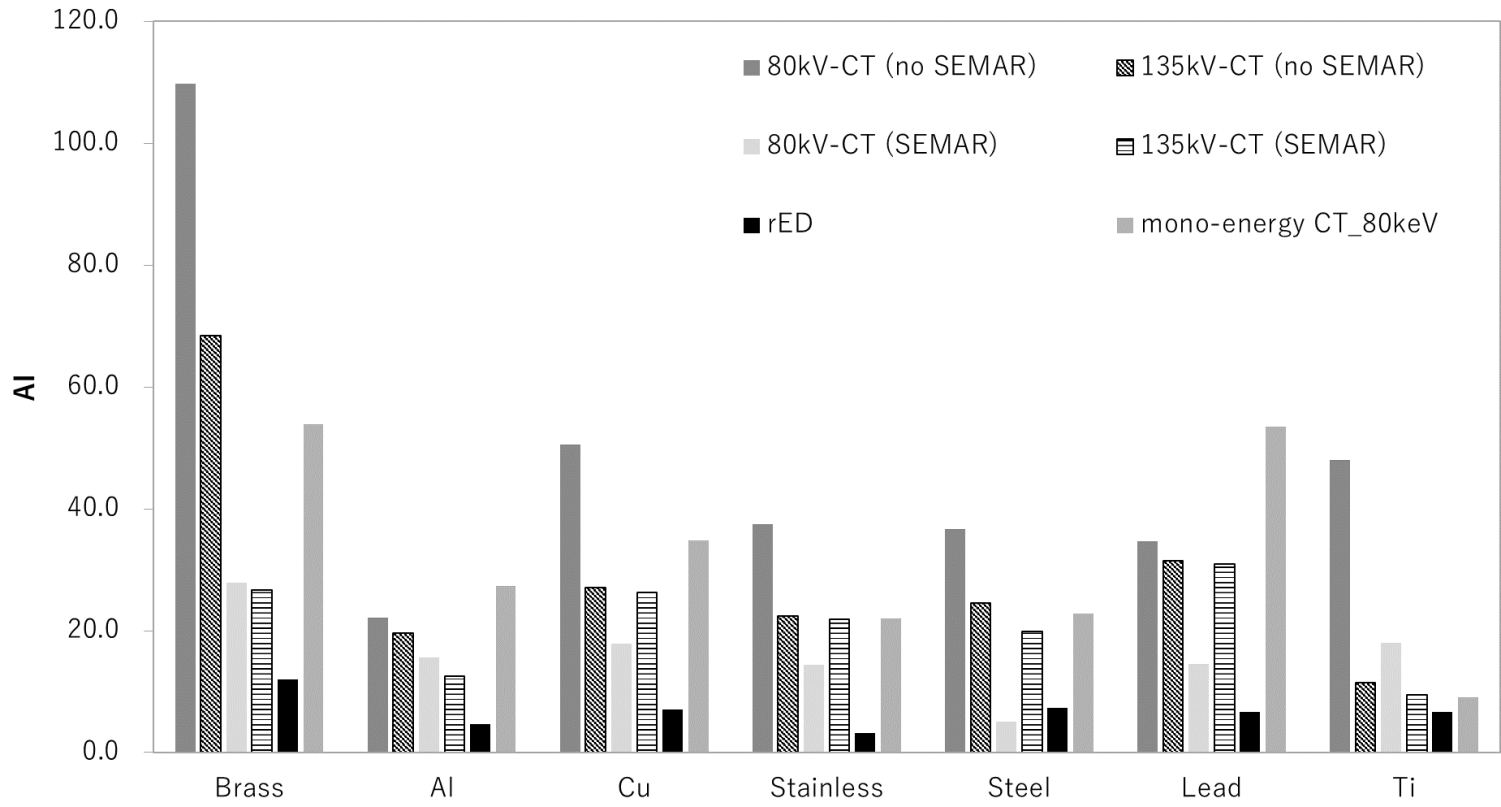
AI of 80 kV and 135 kV CT images with and without SEMAR, mono-energy CT image at 80 keV and rED image in PMMA phantom that inserted metal materials (brass, lead, steel, stainless, copper, aluminum and titanium).

Figure 5 shows the rED, monochromatic CT at 80 keV and kV-CT images with and without SEMAR using brass in the syringe. Beam hardening artefact in the kV-CT images with SEMAR were less than without SEMAR. Although the rED inside the materials was affected from strength beam hardening effects, the metal artefact was caused around the metal insert. The metal artefact in monochromatic CT at 80 keV was less than kV-CT images without SEMAR. Figure 6 shows the AI of the 80 kV and 135 kV CT images with and without SEMAR, the monochromatic CT image at 80 keV, and rED image in the PMMA phantom that inserted metal materials. The AI was smallest for the 80 kV-CT image with SEMAR in steel and the rED image for the other materials. The AI of the kV-CT image with SEMAR was smaller than without SEMAR in all metal materials. From the comparison of 80 kV and 135 kV-CT images with SEMAR, the AI of the 80 kV-CT with SEMAR was smaller than that of the 135 kV-CT image in copper, steel and lead.

### ED-based evaluation for the AI for rED and converted ED images

**Figure 7.**
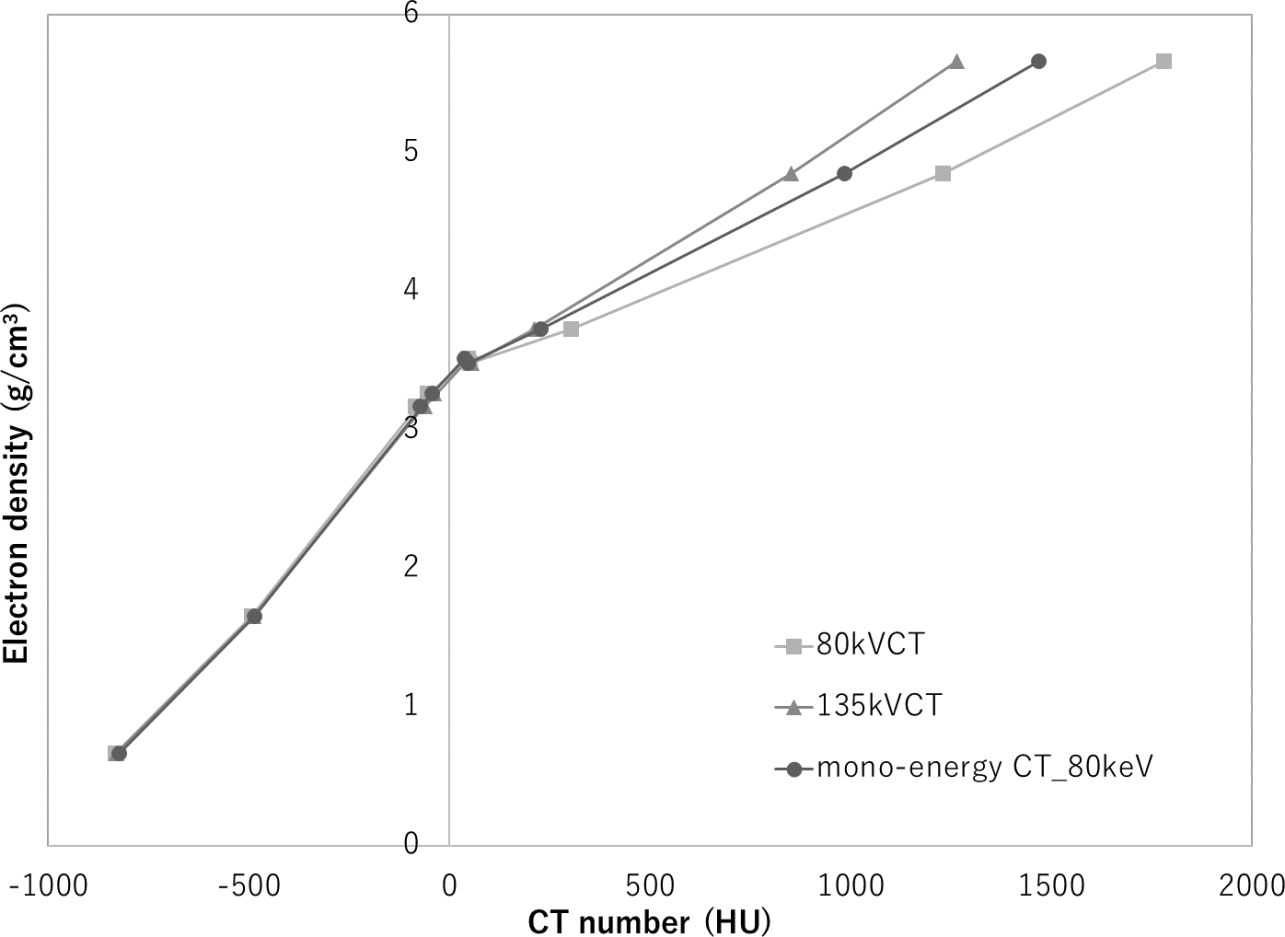
CT-ED calibration table of the 80 kV and 135 kV CT images, and the mono-energy CT image at 80 keV and the rED image for the CIRS 062M phantom.

**Figure 8.**
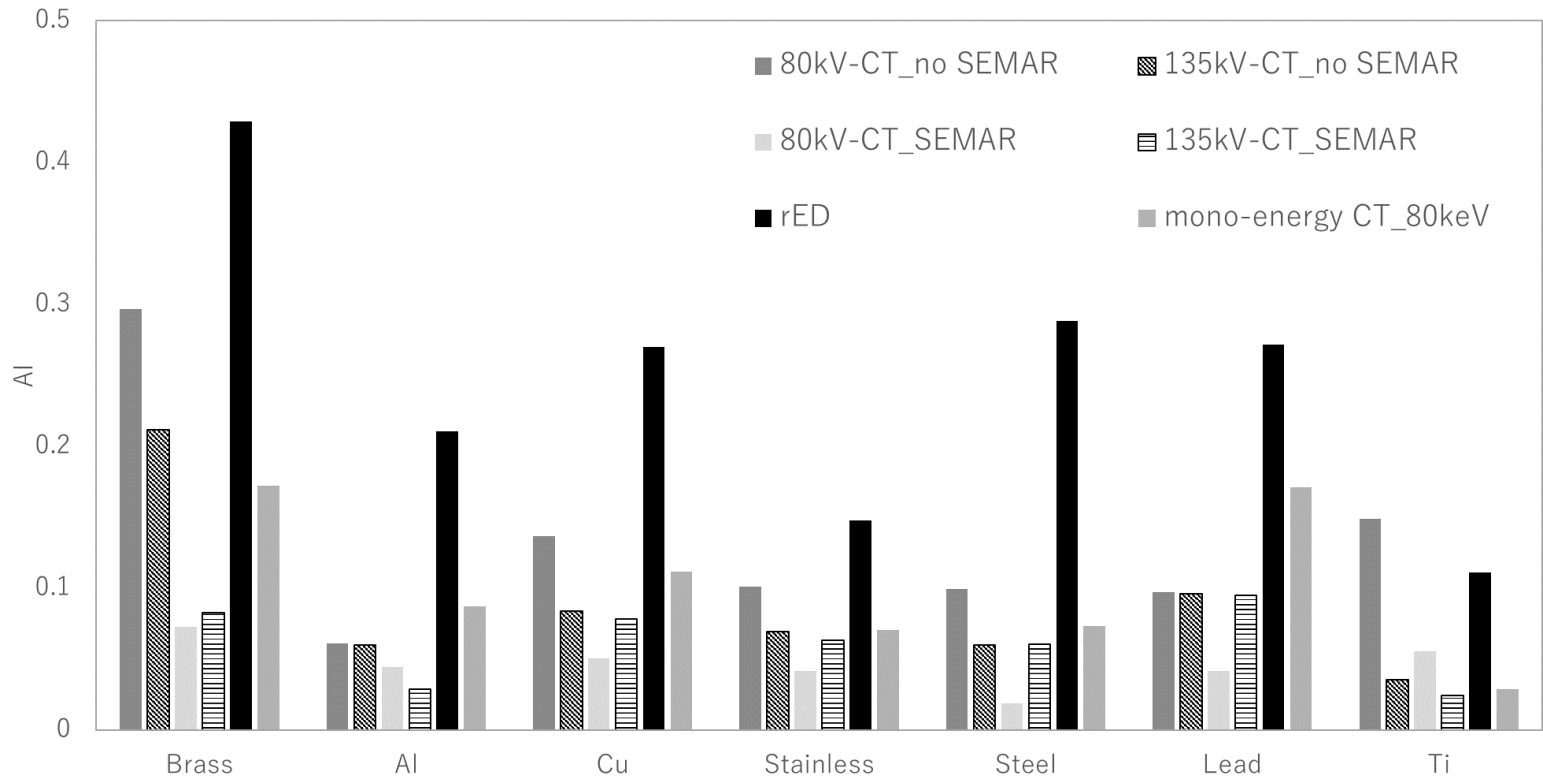
AI of rED and ED images that were converted from 80 kV and 135 kV CT images with and without SEMAR, monochromatic CT image at 80 keV in PMMA phantom that inserted metal materials (brass, lead, steel, stainless, copper, aluminum and titanium).

Figure 7 shows the CT-ED calibration table for 80 kV and 135 kV-CT images and the monochromatic CT image at 80 keV and rED image of the CIRS 062M phantom. Using these calibration tables, the kV-CT and monochromatic CT images were converted to ED images for the PMMA phantom with inserted metal materials. Figure 8 shows the AI for the rED and ED images that were converted from 80 kV and 135 kV-CT images with and without SEMAR and the monochromatic CT image at 80 keV of the PMMA phantom with inserted metal materials. The AI was smallest for the ED converted from the 80 kV-CT image without SEMAR in brass, copper, stainless, steel and lead, and the ED converted from the 135 kV-CT image without SEMAR in aluminum and titanium. In contrast, the AI was largest for the ED converted from 80 kV-CT images without SEMAR in the titanium, and the rED image for the other materials.

## Discussion

The current study showed artefact reduction around various metals in kV-CT images with and without SEMAR by single energy reconstruction, monochromatic CT images and rED images obtained by DECT.

Previous studies have highlighted metal artifacts in patients with hip arthroplasties.^14–17^ These studies have shown a reduction of metal artifacts and an improvement in image quality of soft-tissue structures using SEMAR from Toshiba, compared with FBP. In the single energy reconstruction images, it was revealed that the metal artefact was reduced in all metal materials at both 80 kV and 135 kV using SEMAR. Moreover, the reduction of the AI in the 80 kV-CT image was larger than that for the 135 kV-CT image in all metal materials. Although the metal artefact was reduced for high-energy kV-CT images, the metal artefact was significantly improved with SEMAR for low-energy kV-CT. Especially, the AI of the 80 kV-CT image with SEMAR was smaller than that for the 135 kV-CT image for steel, stainless and lead. Yi, et al evaluated O-MAR in CT orthopedic metal artefact reduction at different tube voltages. An optimal tube voltage was identified for clinical practice and its clinical application was investigated. The AI tended to decrease with an elevated tube voltage at constant mA for both O-MAR and non-O-MAR images.^17^ In the current study, we used the kV-CT images that were scanned based on the DECT scan parameter. The scanned exposures at 80 kV-CT were four times larger than at 135 kV-CT using the DECT protocol. Thus, the metal artefact reduction could depend on the DECT protocol, energy and the scanned materials.

Montner et al found that the errors in the measured CT numbers depended on both the material being imaged and the monochromatic image obtained by DECT.^18^ They reported that the inaccuracy of the measured monochromatic CT numbers was lower at 80–100 keV. Our past study reported that the optimum energy for monochromatic CT for human tissue and iodinated contrast medium is 70–90 keV.^19^ In the current study, the minimum AI value with all metal materials was 80 keV. The results indicated that the monochromatic CT at approximately 70–90 keV could reduce artifacts. Based on this finding, the beam hardening artefact correction could be accurately obtained at 80 keV with the Toshiba DECT. However, a monochromatic CT image that was extrapolated from fast-kilovoltage-switching DECT from GE Healthcare generated at 105 keV, showed superior reduction of metallic artifacts.^20^ The reconstruction technique, effective energy, energy pairs for the DECT scan, and the scan method were different for the different vendors.

From the comparison of the rED image, the kV-CT images with and without SEMAR, and monochromatic CT images at 80 keV, the AI of the rED image was smaller compared with the other images. DECT imaging enables image reconstruction from two different energy pairs and it is possible to create monochromatic CT images. However, the contrast scales in the kV-CT image, monochromatic CT, and rED were different and it should therefore be evaluated using the same table. The current study compared the AI of these different contrast scale images by the measured and converted ED images. From the result of the comparison with the rED image and the converted ED image from the kV-CT images with and without SEMAR, and the monochromatic CT images at 80 keV, the AI of the rED was determined to be larger compared with the other images. In contrast, the AI at 80 kV or 135 kV-CT image with SEMAR was the smallest. The metal artefact reduction methods are different for single energy CT with SEMAR and DECT. The SEMAR technique used in the single-energy CT was applied to raw projection data, such as modified iterative reconstruction (IR) methods and projection interpolation algorithms. The techniques have been shown to be more general and effective for reducing artifacts. The SEMAR algorithm reconstructs and automatically identifies metal traces in sinogram images by applying a HU threshold. The identification of metal segment is performed in the original sinogram through forward projection and removed by neighboring non-metal measurements. The current study compared the ED values converted from the kV-CT and monochromatic CT numbers. Compared to the metal artefact reduction techniques of single energy CT with SEMAR and DECT, the single energy CT with SEMAR could reduce the metal artefact for various metal materials. The metal artifacts that are caused by beam hardening could be reduced by using a monochromatic CT image at high energy. Although the rED was created using the sinograms of the high-kV CT and low-kV CT images, the sinogram based beam hardening correction was not be performed. Thus, the AI in the rED image was larger than that of monochromatic CT at high energy CT and kV-CT with MAR techniques. Niehues, et al also compared the SEMAR and Adaptive Iterative Dose Reduction (AIDR) techniques for the metal artefact reduction^21^. They showed that the SEMAR could contribute to reduce the metal artefact significantly. From above, the SEMAR by single-energy reconstruction would improve the metal artefact and reduce the dose calculation error on radiation treatment planning.

The current study did not evaluate clinical images. A future study should be performed to evaluate image quality due to the metal artefact by hip replacements and sternal fixation in clinical images and the effect of dose distribution in radiation treatment planning.

## Conclusion

The AI was evaluated on the same contrast scale by using the rED and the converted ED from the kV-CT images and the monochromatic CT images. In the comparison of the single energy CT and the DECT techniques, the single energy CT with SEMAR was observed to substantially reduce metal artifacts.
